# Utilizing proteomics to understand and define hypertension: where are we and where do we go?

**DOI:** 10.1080/14789450.2018.1493927

**Published:** 2018-07-12

**Authors:** Christian Delles, Emma Carrick, Delyth Graham, Stuart A. Nicklin

**Affiliations:** Institute of Cardiovascular and Medical Sciences, University of Glasgow, Glasgow, UK

**Keywords:** hypertension, precision medicine, proteomics

## Abstract

**Introduction**: Hypertension is a complex and multifactorial cardiovascular disorder. With different mechanisms contributing to a different extent to an individual’s blood pressure, the discovery of novel pathogenetic principles of hypertension is challenging. However, there is an urgent and unmet clinical need to improve prevention, detection, and therapy of hypertension in order to reduce the global burden associated with hypertension-related cardiovascular diseases.

**Areas covered**: Proteomic techniques have been applied in reductionist experimental models including angiotensin II infusion models in rodents and the spontaneously hypertensive rat in order to unravel mechanisms involved in blood pressure control and end organ damage. In humans proteomic studies mainly focus on prediction and detection of organ damage, particularly of heart failure and renal disease. While there are only few proteomic studies specifically addressing human primary hypertension, there are more data available in hypertensive disorders in pregnancy, such as preeclampsia. We will review these studies and discuss implications of proteomics on precision medicine approaches.

**Expert commentary**: Despite the potential of proteomic studies in hypertension there has been moderate progress in this area of research. Standardized large-scale studies are required in order to make best use of the potential that proteomics offers in hypertension and other cardiovascular diseases.

## 1. Introduction

Blood pressure is a physiological parameter that results from the product of cardiac output and vascular resistance [1,2]. Depending on the requirements of the organism, blood pressure can change within a wide range in order to maintain perfusion pressure of tissues and organs. While blood pressure is typically low during periods of rest and sleep, it can be high during periods of stress and exercise. Blood pressure also varies throughout the day depending on hormonal and other regulatory mechanisms and it shows longer-term variability due to seasonal and other environmental influences [3]. In addition, some organs, including the kidneys and the brain have sophisticated mechanisms to maintain constant perfusion pressures irrespective of systemic blood pressure [2]. These phenomena have been subject to in-depth studies in the systemic circulation whereas our knowledge of blood pressure regulation in other vascular beds, for example the pulmonary circulation is less far developed – in part due to the difficulties in measuring pulmonary arterial pressure other than by invasive methods.

It is apparent that this seemingly simple physiological parameter is much more complex than originally thought. This complexity has implications for the definition of ‘normal’ and ‘high’ blood pressure where clearly an isolated reading at a random time of the day cannot be used to establish the diagnosis ‘hypertension’ [4]. In fact, hypertension is different from many other clinical conditions whose definition is primarily based on the underlying pathology, possibly combined with diagnostic criteria based on symptoms and test results. In contrast, hypertension is exclusively defined on a readout, and while possible underlying mechanisms are acknowledged, they are not part of its definition.

Unsurprisingly, the definition of hypertension has therefore changed over the years. Where the potential harmful consequences of high blood pressure have not been generally accepted up until less than 100 years ago [5], the association between high blood pressure cardiovascular damage and reduced lifespan became apparent with the availability of large epidemiological data, such as the Framingham Heart Study [6]. Definitions of hypertension have been based on systolic, diastolic or both pressures and have generally seen a higher cut-off in the past and much lower thresholds in more recent years. While most guidelines still define hypertension as blood pressure above 140 mmHg systolic and/or 90 mmHg diastolic [7–9], the most recent editions of clinical guidelines now propose 130/80 mmHg [10], mainly driven by a contemporary randomized clinical trial that provides evidence for better outcome with lower blood pressure targets [11]. It is important to note, however, that research data, as well as guidelines acknowledge the fact that there is a continuous relationship between blood pressure level and cardiovascular risk [12] and that thresholds and dichotomous categorizations in the first instance serve as clinical decision tools rather than reflecting biological mechanisms. As such, the thresholds to define hypertension are driven by a number of factors, including risk and benefit of treatment, health economic aspects, and of course trial evidence.

**Figure 1.**
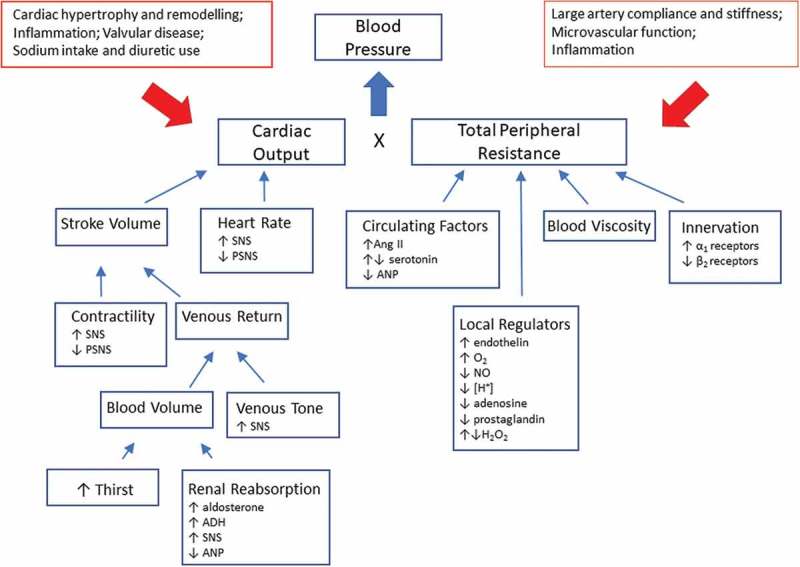
**Key principles of blood pressure regulation**. Blood pressure is the result of cardiac output and peripheral resistance where a multitude of factors contribute to these two components. The figure illustrates some of these principles that are subject to any studies into the pathophysiology of hypertension. ADH, antidiuretic hormone; Ang II, angiotensin II; ANP, atrial natriuretic peptide; PSNS, parasympathetic nervous system; SNS, sympathetic nervous system.

**Figure 2.**
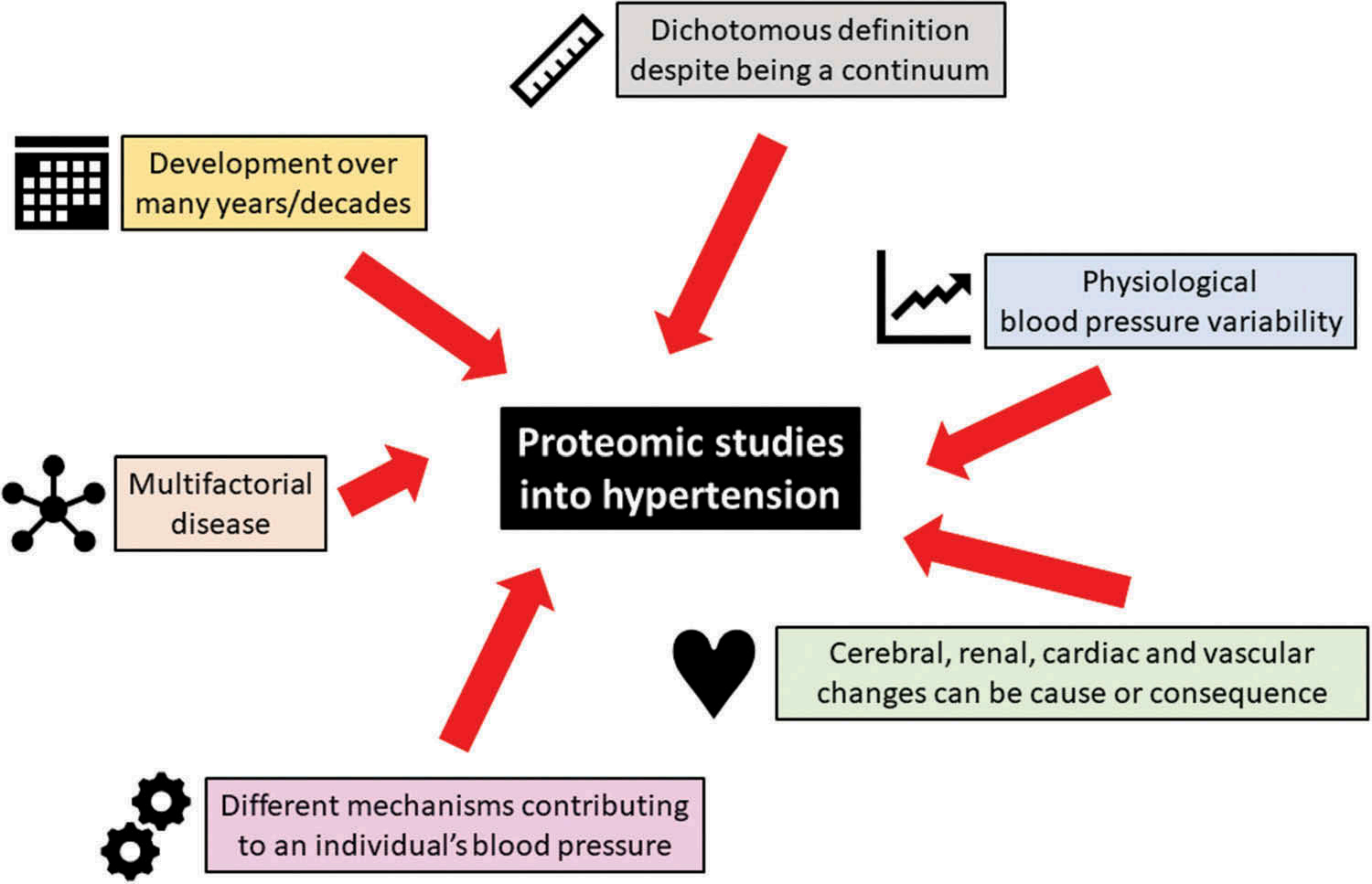
Challenges in hypertension that impact on proteomic studies.

Even if blood pressure is a result of cardiac output and vascular resistance each of these factors can have multiple modulators (Figure 1). In a minority of people with hypertension a single distinct factor, such as activation of the renin angiotensin aldosterone system (RAAS) by renal artery stenosis, or production of excess aldosterone in primary aldosteronism drives their high blood pressure (secondary hypertension), whereas the majority of patients have primary hypertension where multiple dysregulated pathways together contribute to high blood pressure. It is evident that any research into the origins of primary hypertension will result in the discovery of multiple reasons and that by definition this condition will have complex origins. Evidence from genome-wide association studies has shown that despite the discovery of a large number of genetic variants that are robustly associated with blood pressure regulation or hypertension their absolute contribution to the phenotype remains small [13]. A complex disease with multiple causes that is to some extent arbitrarily defined based on a blood pressure threshold is not an easy target for research (Figure 2). In this article we will explore whether current strategies to unravel the origins of hypertension can benefit from proteomic approaches. Similar to traditional approaches we will also employ a reductionist approach and focus on well-defined animal models before we explore the use of proteomics in human hypertension. We would like to emphasize that we cannot provide a systematic review of all studies in various models of hypertension or clinical conditions associated with hypertension. Instead we will highlight some key papers to illustrate the development and future potential of proteomics in hypertension.

Hypertension and associated cardiovascular diseases are a global health burden with increasing prevalence across all continents [14–16]. Hypertension is in many cases asymptomatic and awareness of the condition is therefore limited until organ damage, such as stroke, myocardial infarction, or heart failure occur. However, even those who are aware of high blood pressure are often not receiving treatment, and blood pressure control rates in treated patients remain low [16,17]. It is evident that in addition to widespread use of existing diagnostic and therapeutic options there must be novel approaches to tackle this global problem.

## 2. Proteomics

We cannot provide a comprehensive overview of proteomic techniques in this review but would like to briefly put experimental and clinical data into the context of contemporary proteomic techniques. Readers are referred to review articles providing further background, including a recent American Heart Association statement [18]. The term proteomics was first coined in 1994 by Marc Wilkins as the study of an organism’s proteome at a given time [19]. Techniques involved in proteomics include, but are not limited to, sodium dodecyl sulfate polyacrylamide gel electrophoresis (SDS-PAGE), 2D-difference gel electrophoresis (2D-DIGE), immunoassays, protein microarrays, and variations of mass spectrometry. 2D gel electrophoresis was used to observe that ferritin light chain was significantly increased in coronary artery disease [20]. 2D protein gel databases for human and other animal models have been created and used for reference [21].

The most common analytical technique used today is nanoflow liquid chromatography coupled to a mass spectrometer (nLC-MS). The development of nano electrospray in 1996 by Wilm and Mann [22] increased the sensitivity of the analysis of biomolecules to a point were it was possible to analyze more complex samples. Despite this, a limitation of proteomic analysis by mass spectrometry is dynamic range; an MS is only able to select a limited amount of ions present in a scan for further fragmentation (MS/MS), and these ions are selected on abundance. Therfore, proteins that are of interest are often not identified in complex systems. Throughout the development of mass spectrometers advances have been made in the speed of analysis allowing for an increase in the number of MS/MS events permitted in a duty cycle. It is not uncommon today for the 20 most abundant ions in every scan to be selected for MS/MS. However, this still means that there are many ions not selected for MS/MS and therefore a chance at identification is lost.

Since the early 2000’s, there has been a steady stream of technological advances that have enabled researchers to dig further into the proteome. Subsequently, proteomics has moved from a gel approach to a solution-based analysis. The extraction of proteins and the preparation of samples and their subsequent digestion to peptides in solution is the most popular form of proteomics analysis at present. The development of Filter-Aided Sample Preparation (FASP) combines the benefits of both in-gel and in-solution digestion strategies [23]. The addition of SDS and urea facilatates a denaturing effect for gel electrophoresis and facilitates the solubilization of cells and tissues. FASP has enabled researchers to prepare vascular tissues socu as for proteomic analysis simply and relatively quickly [24]. Advances in samples preparation have gone hand in hand with advances in instrumentation. With the advances in quadrupole and time of flight (TOF) analyzers, which have been the mainstay of proteomic mass spectrometry since its inception, there was the introduction of new analyzers in the early 2000’s. The Orbitrap MS was a jump in sensitivity, mass accuracy, and resolution [25]. The original Orbitrap hardware was continually improved and enhanced and by the time FASP was developed the LTQ Orbitrap Velos could routinely perform 20 MS/MS per second. However, it is important to remember that there is not one LC-MS platform that can cover the entire proteome; the data generated from any of the many platforms are wholly reliant on experimental design.

There are two major approaches to proteomic analysis the shotgun approach were samples are digested by a proteolytic enzyme, usually trypsin, followed by nLC-MS/MS. In this approach the methods are optimized for peptide separation and chromatographic peak resolution. The other method is a targeted approach where the mass spectrometry analysis is targeted to the *m*/*z* of the known compound or peptide. This analysis is only possible for the monitoring of specific known analytes. For optimizing targeted approaches, it is not uncommon to employ a standard of the known analyte in order to gain information on the endogenous analyte, e.g. charge state and retention time [26]. In cardiovascular disease both approaches have been deployed to lead to a better global understanding of coronary artery disease [27]; or the rapid targeted analysis of the concentrations of angiotensin II (Ang II), Ang-(1–7), Ang III, and Ang IV [28]. In addition it is also possible to analyze native peptides, where no digestive enzyme is used in the preparation, such as the diagnostic biomarkers present in urine for coronary artery disease [29,30].

Protein quantitation studies enable researchers to analyze the changes in protein expression levels in response to pharmacological or other stimuli or disease states. Methods of protein quantitation include *in vitro* methods, such as isobaric tags for relative and absolute quantitation (iTRAQ) and tandem mass tags (TMT) where samples are tryptically digested and labeled with a commercial tag. The samples are then mixed and analyzed by nLC-MS/MS. In terms of protein labeling, Stable Isotope Labeling with Amino acids in Cell culture (SILAC) is the only metabolic *in vivo* protein quantification method. Cells or very rarely animals are fed a heavy labeled isotope; the experimental conditions are therefore labeled as light, medium, or heavy from the outset. The samples are then mixed and prepared as one sample. Peptide quantification is achieved on the ions before fragmentation for peptide identification [31].

None of these techniques, however, are possible without the use of bioinformatics. In global shotgun analysis the spectra that are acquired are just data. There are a multitude of software packages both commercially available (e.g. Proteome Discoverer, Protein Pilot, Progenesis) and open source (for example Max Quant and the Trans-Proteomic Pipeline (TPP)) that enable proteomic workflows to be applied to the analysis of the wide variety of studies, including those in the cardiovascular field.

While proteomic techniques have found their way into the study of some cardiovascular diseases [18,32], the number of applications in hypertension remain limited. The above mentioned limitation of some of the techniques, the dependence of results on the type of biosamples and proteomic platforms used, and the heterogeneity of hypertension as a clinical condition are some of the hurdles that have to be tackled. In order to reduce the pathophysiological complexity of hypertension, researchers are using rodent models with well characterized forms of htypertension. We will provide an overview of proteomic studies in two specific models: the Ang II infusion model of hypertension and the spontaneously hypertensive rat (SHR).

## 3. Experimental models of hypertension

Many of the primary and secondary models of hypertension that are commonly used in the study of hypertension have a dysregulated RAAS, which contributes to the increased blood pressure and end organ damage, for example, the two kidney one clip model [33] and SHR [34]. However, these models are complex with hypertension and associated end organ damage developing through the interplay of a myriad of risk factors, including genetic influences, or the complications of mechanical injury following surgical intervention. An experimental model that enables the study of Ang II in isolation would facilitate proteomics studies that could directly investigate target pathways involved in hypertension in response to Ang II. The Ang II infusion model of hypertension is exactly such a model.

We will therefore present a number of proteomic studies that used the Ang II infusion model to dissect specific aspects of hypertension and other cardiovascular diseases. In addition, we will review studies that have been conducted in the SHR, a widely used model of human essential hypertension and its cardiovascular sequelae. We are aware that there are numerous other preclinical models of hypertension but the choice of these two models will illustrate the spectrum of possibilities ranging from specific pathways (Ang II) to complex genetically determined hypertension (SHR).

### 3.1. The Ang II infusion model of hypertension and end organ damage

The RAAS regulates blood pressure through a combination of mechanisms influencing renal electrolyte handling, vascular smooth muscle cell (VSMC) vasoconstriction, aldosterone release, and effects on thirst and water preservation. Renin is released into the bloodstream and cleaves angiotensinogen to produce Ang I, which is subsequently cleaved by angiotensin converting enzyme (ACE) to generate Ang II [35]. Ang II has two main receptors, the angiotensin type 1 and type 2 receptors (AT_1_R and AT_2_R, respectively). Predominant effects of Ang II are mediated via the AT_1_R, with the AT_2_R being largely absent in normal adult tissues. Chronic RAAS activation leads to the development of hypertension and subsequent secondary end organ damage through pressure overload in tissues, such as the heart and kidney leading to cardiac hypertrophy and fibrosis. Moreover, since the AT_1_R is ubiquitously expressed Ang II can have direct effects on cardiomyocytes and fibroblasts, further contributing to end-organ damage [36,37]. Therapies targeting Ang II generation (ACE inhibitors) or Ang II action (angiotensin receptor blockers; ARBs) are mainstay treatments to reduce blood pressure and to reduce end organ damage and subsequent morbidity and mortality in heart failure post-myocardial infarction.

The infusion model of Ang II is a method for studying the role of Ang II in blood pressure regulation, the development of hypertension and end organ damage in isolation and has been used in a wide range of rodent models in both mice and rats. Normal healthy animals are subcutaneously implanted with osmotic mini pumps that constitutively and chronically infuse Ang II at a defined dose [36,38]. The dose of Ang II is important to determine the type of hypertension that develops with sub-pressor and pressor dose models developed. Although the dosing of Ang II is hugely variable in the literature and in different models, sub-pressor doses (typically up to 200 ng/kg/min in rats administered subcutaneously [38]) produce a gradual increase in BP over a period of approximately 1 week and peaking at 2 weeks without renal dysfunction or sodium or water retention [39]. In this model, the blood pressure increases are thought to arise due to a combination of direct VSMC vasoconstriction and oxidative stress. In contrast, infusion of pressor doses of Ang II (greater than 200 ng/kg/min delivered subcutaneously in rats [38]) leads to acutely increased BP in a matter of minutes to hours and involves secretion of aldosterone, retention of water and sodium, resetting of baroceptors, and increased sympathetic output [38]. The use of different doses of Ang II in these models therefore offers the opportunity to compare and contrast different pathways by which hypertension and end organ damage develop and to delineate the effects of one molecule at a multi-system level in an *in vivo* model. Using both sub-pressor and pressor doses of Ang II also leads to direct effects, which mediate target end organ damage, including direct promotion of cardiomyocyte hypertrophy, vascular hypertrophy, and cardiac and renal fibrosis. The Ang II infusion model is therefore useful for the study of hypertension and end organ damage specifically in response to Ang II. By only altering one factor in recipient animals the many different pathways regulated by Ang II including vascular tone, tissue remodeling, inflammation and oxidative stress can be studied. Although these models may not recapitulate all of the complexities of essential hypertension in patients, nonetheless they offer useful models to facilitate clearer understanding of specific RAS-related pathways.

#### 3.1.1. Proteomic studies in the Ang II infusion model of hypertension and end organ damage

Relatively few studies to date have utilized the Ang II infusion model to study proteomic changes in relation to the role of Ang II in hypertension. Using a rat model of Ang II infusion, a direct comparison of the effects of both pressor (60 ng/min) and sub-pressor (15 ng/min) doses in renal proximal tubules via phosphoproteomic screening was performed [40]. This led to profiling of 38 phosphoproteins, including 14 that were only significantly altered via the pressor doses, highlighting differences in the response to sub-pressor and pressor doses of Ang II. Increased phosphorylation of protein kinase C (PKC) isoforms (PKCΦ061 and PKCβII), as well as glycogen synthase kinases (GSK) 3Φ061 and β were detected. Conversely, PKCσ and cAMP response element phosphorylation was decreased. Following infusion of sub-pressor Ang II doses only seven phosphoproteins were altered, including increased PKCα, PKCσ and GSKα phosphorylation. These effects were sensitive to blockade of the AT_1_R with the ARB losartan, and the data implicated roles for PKC, GSK and cAMP signaling being important in the renal response to Ang II infusion. Furthermore, utilizing AT_1_R knockout mice in comparison to wild type mice, proteomics was used to demonstrate that in the kidney of AT_1_R knock-out mice the thiazide-sensitive co-transporter and amiloride-sensitive Na^+^ transporter were downregulated while the epithelial sodium channel (ENaC) isoforms γ and β were significantly increased [41]. These studies confirmed the importance of the AT_1_R in regulating sodium transporters and ion channel proteins in the kidney. These two publications highlight the important role of Ang II signaling via the AT_1_R to regulate the renal response to changes in electrolytes and fluid volume to maintain BP.

Using Fisher rats infused with a pressor dose of Ang II (450 ng/kg/min) for 14 days the platelet proteome was investigated based on prior knowledge that hypertension is a risk factor for arterial thrombosis [42]. In isolated and purified platelets 45 proteins, including talin-1, thrombospondin-1, transgelin-2, and filamin-A were determined to show hypertension-associated changes. All the proteins were normalized within 10 days of Ang II withdrawal and animals returning to normotension. The authors concluded that these proteins may constitute useful biomarkers for monitoring BP. Using Ang II infusion in mice (50 ng/min), it was demonstrated that during the development of hypertension high resolution proteomics could be used to identify protein aggregates in the heart [43]. Identified proteins included ApoE, ApoJ, ApoAIV, clusterin, and complement C3, all proteins previously implicated in aging-associated fibrosis or aging-associated neurodegenerative conditions. These findings highlighting that diverse disease processes associated with fibrosis lead to changes in protein aggregates, defining common proteomic changes that may be associated with disease development. The effects of caloric restriction on Ang II–induced cardiac damage and mitochondrial dysfunction was investigated using a double transgenic rat expressing renin and angiotensinogen genes that leads to endogenous elevated Ang II levels and hypertension [44]. In this model, proteomic profiling of mitochondria isolated from the hearts revealed changes in proteins associated with cytoskeletal rearrangement, and oxidoreductase activity and provided evidence that calorie restriction could protect the mitochondria from Ang II-induced damage and dysfunction.

#### 3.1.2. Proteomics studies of the RAAS in cardiovascular cell culture models

Proteomic screening has also been utilized in *in vitro* primary cell models related to hypertension to refine understanding of the effects of Ang II on individual cell types. This may offer advantage of simplifying data analysis and interpretation since despite the Ang II infusion model only relying on altering levels of one molecule, the pleiotropic effects of Ang II on a number of different pathways in a range of different organs during the development of hypertension highlights that it still results in the generation of complex data sets. Using VSMC the Ang II-regulated secretome was analyzed via proteomics [45]. In conditioned media from VSMC stimulated with Ang II 629 proteins were identified, with 26 being differentially expressed between Ang II and control stimulated cells. Of the proteins identified several were related to growth including clusterin and growth arrest-specific 6, and were decreased in response to Ang II. Other identified proteins including cathepsin B, plasminogen activator inhibitor-1 (PAI-1) and periostin were also demonstrated to be differentially regulated.

An endogenous counter-regulatory axis of the RAAS exists centered on an ACE homologue ACE2 and generation of the active peptide hormone Ang-(1–7), which acts at the receptor Mas and can antagonize pathophysiological effects of Ang II at the AT_1_R (reviewed in McKinney et al. [46]). Using *in vitro* cell culture models the first studies into the counter-regulatory axis of the RAAS have been performed to identify novel signaling mechanisms of the peptide hormone Ang-(1–7) [47]. The phosphoproteome of Ang-(1–7)-stimulated human aortic endothelial cells was investigated and identified novel Ang-(1–7) targets, including forkhead box protein O1 and proline rich AKT1 substrate 1. Furthermore, the studies highlighted changes in phosphorylation of downstream effectors of the insulin signaling cascade, revealing a potential novel role for Ang-(1–7) in regulating glucose homeostasis in endothelial cells. A more recent study, also in human aortic endothelial cells, revealed that Ang-(1–7) stimulated cofilin-1 expression [48]. Cofilin-1 is known to be involved in regulating actin filament dynamics and in cytokinesis [49] and has previously been implicated in cancer progression [50]. Ang-(1–7) stimulated G0/G1 arrest through a pathway involving cofilin 1, suggesting its importance for maintaining cell cycle homeostasis in response to Ang-(1–7) [48].

In summary, the role of the RAAS and Ang II in the development of hypertension in patients is well known and the mechanisms are well conserved between humans and rodent models. The development of the Ang II-infusion model of hypertension has offered the opportunity to study the role of Ang II in hypertension in isolation of other confounding factors, such as environment, diet, and idiopathic genetic influences. However, the broad range of concentrations of Ang II utilized in studies across different timepoints, combined with the range of effects of Ang II in different tissues means data interpretation is complex. Although *in vivo* studies have highlighted potential new molecules regulated by Ang II, many of the general signaling pathways had already been identified by targeted research. In *in vitro* models more specific questions can be asked and less complex data sets have revealed novel signaling pathways in both the classical RAAS and the counter-regulatory RAAS axis. Future research is required to fully understand how these novel pathways contribute to regulation of the RAAS *in vivo* in the development of hypertension and to translate these findings into the patient.

### 3.2. Spontaneously hypertensive rats as model of human primary hypertension

The SHR is an inbred strain that is by far the most widely studied model of human essential hypertension. The strain was developed in Kyoto in the 1960s by selective inbreeding for high blood pressure from outbred Wistar rats [51]. The SHR is characterized by a number of phenotypic abnormalities, including spontaneous hypertension, cardiac hypertrophy, and vascular dysfunction [52]. Blood pressure begins to rise between 5 and 6 weeks of age in SHR and becomes fully established around 15–20 weeks of age [52]. The closely related stroke prone SHR (SHRSP), displays more severe hypertensive phenotype than the SHR, in addition to spontaneous strokes and salt sensitivity [53,54].

#### 3.2.1. Proteomic studies in the spontaneously hypertensive rat

SHR have been extensively characterized at both the genomic and phenotypic level [55–57], and the model has been used to examine the proteome in a range of cardiovascular tissues. Examples include a study by Jin et al. [58] that examined protein expression changes occurring during development of left ventricular hypertrophy, using 2D gel electrophoresis in combination with matrix assisted laser desorption/ionization – time of flight tandem mass spectrometry (MALDI-TOF/TOF MS/MS) in left ventricular myocardium from SHR and normotensive Wistar Kyoto (WKY) rats. They reported 13 proteins that were differentially expressed in the SHR before the onset of hypertension. These proteins included □-enolase and lactate dehydrogenase B, which are two important enzymes for glycolysis, as well as proteins associated with mitochondrial oxidative phosphorylation, oxidative stress and cellular energy metabolism [8]. In addition, a study by Lee et al. [59] also reported the use of 2D gel electrophoresis and MALDI-TOF/TOF MS/MS to study protein expression profiles in aortic smooth muscle of SHR and WKY rats. They identified seven proteins that were differentially expressed between the strains, including reduced expression of dihydropteridine reductase (DHPR), which is associated with the regeneration of tetra-hydrobiopterin (BH_4_) and is involved in the development of vascular oxidative stress [60]. A recent study by Hatziioanou et al. [61] that used 2D gel electrophoresis of renal parenchyma from SHR and WKY rats identified overexpression of chloride intracellular channel 4 (CLIC4) protein in the proximal tubule during early stages of hypertension development. This finding suggests that CLIC4 may be a useful early marker to detect renal tubular alterations in the development of hypertensive nephrosclerosis.

The SHR has also been used to examine proteome impact after pharmacological or physical intervention. For example, proteomic changes occurring in the aorta as a result of physical activity-induced blood pressure reduction have been demonstrated [62]. In this study, young SHR and WKY rats were exposed to a 6-week load-free swimming program, which significantly reduced blood pressure in the hypertensive model. Proteomic analysis was carried out by LC-MS in aorta from exercise-trained and sedentary SHR and WKY rats. When compared with sedentary SHR rats, nine differentially expressed proteins were identified, five were up regulated (long-chain specific acyl-CoA dehydrogenase, heat shock protein β-1, isocitrate dehydrogenase subunit α, actin, α cardiac muscle 1 preprotein, and calmodulin isoform 2) and four were down regulated (adipocyte-type fatty acid-binding protein, tubulin β-2C chain, 78 kDa glucose-regulated protein precursor, and mimecan). This study confirms that proteomics is an effective method to identify the target proteins of exercise intervention for hypertension.

#### 3.2.2. Beyond proteomics: multilevel omics and integration of genetic information

A study by Low et al. [63] combined proteomics with in-depth genomic and transcriptomic analysis allowing a multilevel systems approach for identification of candidate genes involved in the SHR hypertensive phenotype. In this study, the liver proteome was compared between a specific subline of the SHR (SHR/Olalpcv) and the Brown Norway rat (BN-Lx). This multilevel approach identified 26,463 rat liver proteins, and validated 1,195 gene predictions, 83 splice events, 126 proteins with nonsynonymous variants, and 20 isoforms with nonsynonymous RNA editing. A major finding of this study was the identification of a genomic variant in the promoter of the Cyp17a1 gene, which is a previously reported top hit in human genome wide association studies for hypertension.

In addition to examining tissue proteome differences between inbred normotensive and hypertensive strains, there is huge potential for studying downstream effects of specific genetic modifications. Studies of genetically modified rats can be used to reduce the complexity of the proteomics analysis, where a single gene or a restricted number of genes are responsible for the observed phenotypic differences. An example of this reductionist approach is the recent study by Manakov et al. [64], which compared the cardiac proteome of SHR and transgenic SHR-Cd36 rats, carrying a copy of the wild type CD36 gene. Protein expression profiling was based on two-dimensional gel electrophoresis (2DE) coupled to tandem mass spectrometry and label-free LC/MS in the left and right heart ventricles of SHR and SHRCd36 rats. In total, 26 differently expressed myocardial proteins were identified, out of which 18 were found in the right ventricles and 8 in the left ventricles. This is the first report to reveal an impact of transgenic expression of CD36 on the heart at the proteome level, identifying down regulation of respiratory chain enzymes that suggests a shift in regulation of energy metabolism.

Although the proteome is several orders of magnitude more complex than the genome, it is vital for determining how proteins interact as a system and for understanding the function of cellular systems in disease. This snapshot of proteomics studies in the SHR rat demonstrates the important role that this model plays in understanding the mechanisms underlying hypertension and its end-organ damage.

## 4. Human studies

### 4.1. Proteomic studies in human hypertension

We have already outlined that primary hypertension is a complex cardiovascular condition that is defined by a physiological parameter (blood pressure above a somewhat arbitrary threshold) and not by underlying pathophysiological principles, which can indeed be different between patients. This complexity and heterogeneity of hypertension makes studies into the pathogenesis of hypertension particularly difficult. In contrast to proteomic approaches, genetic or genomic studies can be done at any point in time as the genome remains stable despite changes in the phenotype. With hypertension being mainly a disease of the elderly [65], this is an advantage of genetic over proteomic approaches as genetic variants can already be detected at young age and predict the development of hypertension later in life. However, proteomic studies have the advantage of describing the current state of an organism or its organs or tissues and thereby integrate genetic and environmental factors. These considerations have already been discussed previously by us [66,67] and others [68].

For the above reasons, there are very few studies that define proteomic signatures of hypertension. An exemption is a recent study Gajjala et al. [69] who employed a complex proteomic workflow to discover plasma peptides that are differentially expressed between normotensive and hypertensive subjects. A proteomic score has been developed to differentiate between patients and controls and includes a total of 27 molecular determinants, such as phosphoinositide 3-kinase regulator 1, osteocalcin, and nexilin. Literature mining and pathway analysis confirmed the involvement of these features in atherogenesis, cellular calcium metabolism, cytoskeletal organization, angiogenesis, and other key pathways that are likely to play a role in the pathogenesis of hypertension. The authors discussed that their analysis could not determine whether the observed differences were a cause or consequence of hypertension and referred to other studies that face the same challenges [42,70].

Interestingly, a recent study by Johansson et al. [71] employed targeted proteomic profiling using the Proximity Extension Assay technique to study another blood pressure-related condition: orthostatic hypotension. Plasma proteins that were found to be differentially expressed between patients and controls included matrix-metalloproteinase-7 (MMP-7), T-cell immunoglobulin, and mucin domain-1 (TIM-1) and thereby proteins involved in matrix degradation and inflammation – molecular features that have also been found in studies into the pathophysiology of hypertension. It is reasonable to assume that information about blood pressure regulation can be obtained from proteomic studies at both ends of the extreme, hypertension, and hypotension.

### 4.2. Proteomics and hypertension-associated organ damage

As changes in the proteome directly relate to changes in organ structure and function, proteomic techniques have been used to study organ damage as a result of hypertension. Hypertension affects a variety of organs including the brain, kidneys, eyes, and large vessels and particularly the heart. Using capillary electrophoresis-mass spectrometry (CE-MS) Kuznetsova et al. [72] have established urinary proteomic profiles that are associated with left ventricular diastolic dysfunction, one of the earliest functional markers of hypertensive heart disease. The urinary peptide markers have been further shown to be differentially regulated also in patients with overt heart failure, indicating a molecular pathophysiological continuum from subclinical damage to overt disease [72]. These data have been further confirmed in a larger study in the general population demonstrating correlations between left ventricular dysfunction and the urinary proteome [73]. The urinary proteome can also provide information on cardiovascular endpoints in hypertensive patients where it has been found to predict coronary events in a substudy of the Anglo-Scandinavian Cardiac Outcomes Trial (ASCOT) [74]. A more detailed review of the potential of proteomic studies in cardiac remodeling has been published elsewhere by Petriz and Franco [75].

Similar studies have been conducted in hypertensive patients at risk of developing renal disease. Plasma proteomic studies using LC-electrospray-trap MS have identified molecular signatures based on signaling molecules, growth factors, and angiogenesis factors that are predictive of renal disease in patients with hypertension and/or diabetes [76]. A comprehensive proteomic profiling study in 123 hypertensive patients with chronic RAAS suppression has found signatures that predict development of *de novo* development of albuminuria or indicate stable albuminuria [77]. The signatures were composed of proteins involved in inflammation, immune, and proteasome activation/endoplasmatic reticulum stress. Further studies by these authors in urine samples [78] and urinary exosomes [79] confirmed the link between inflammation and development of albuminuria in people with controlled hypertension on RAAS blockade. The complementary nature of these two studies using different sample types increases the reliability of the data and paves the way to further studies that can explore effects of therapeutic strategies on proteomic signatures of immune dysregulation that will translate into prevention of albuminuria and ultimately, renal failure in patients with hypertension.

### 4.3. Proteomic studies in preeclampsia

While the number of proteomic studies in primary hypertension is relatively small and often focusses on studies into hypertension-associated organ damage there are larger amounts of data available in hypertension in pregnancy and especially in pre-eclampsia. These conditions are particularly attractive research areas for a number of reasons. First, they have an important impact on health with preeclampsia being a main reason for maternal and fetal morbidity and mortality. Second, these conditions develop within the timeframe of pregnancy with a defined start and stop point within less than 9 months and can thereby be studied in their wholeness as opposed to primary hypertension where development spans many decades. Third, preeclampsia develops in previously healthy women and the changes in blood pressure and the related changes in organs, such as the kidney can be easily contrasted to women with normal course of pregnancy. While exact mechanisms of hypertension in pregnancy may be different from those in essential hypertension, it can be reasonably assumed that basic principles will overlap and that data from hypertension in pregnancy will also inform research into other cardiovascular conditions.

Another advantage of research into hypertension in pregnancy is that these conditions, especially preeclampsia, are thought to be driven by placental factors. Access to placenta tissue post partum for molecular studies is much easier than access to other human cardiovascular tissues. For example, Zhang et al. [80] have used MS studies in placentas of women with preeclampsia and controls with normal pregnancies to describe proteins and protein networks that are involved in cellular movement, development, growth, and proliferation. Using a similar study design and a 2D LC-MS/MS quantitative proteomics strategy, Jin et al. [81] have demonstrated a number of differentially expressed proteins between preeclamptic and normal placentas involved in inflammation and defence against oxidative stress. In particular, alterations in proteins involved in glutathione metabolism have been found to be characteristic of placentas from preeclamptic women. A recent study by Mary et al. [82] demonstrated differential regulation of haptoglobin and hemopexin between preeclamptic and normal placentas. In contrast to these studies in whole placenta, Ma et al. [83] have used trophoblast cells obtained by laser capture microdissection and demonstrated differences in 169 out of 831 proteins identified by 2D LC-MS/MS of which downregulation of laminin in trophoblastic cells of women with preeclampsia was the most prominent finding.

A number of studies have used proteomic approaches in biofluids, such as blood and urine to identify markers that could predict the development of preeclampsia. Of these the study by Myers et al. [84] who employed an integrated approach of unbiased screening, targeted quantitation and supplementation of models with known pathogenetic factors to describe novel biomarkers for prediction of preeclampsia. The most promising models centered on insulin-like growth factor acid labile subunit. A urinary proteomic study by Chen et al. [85] employed iTRAQ labeling coupled with 2D LC-MS/MS and identified 113 differentially expressed proteins between women with preeclampsia and those with normotensive pregnancies. Urinary angiotensinogen has been found downregulated in women with preeclampsia pointing toward the critical role of the RAAS in the development of this condition. Carty et al. [86] conducted a CE-MS-based study and described urinary proteomic profiles in first trimester that were different between women who later developed preeclampsia and those who continued to be normotensive; the initial discoveries were, however, not validated in an independent validation cohort.

## 5. Precision medicine and the role of proteomics in hypertension

We have demonstrated that proteomic studies have the potential to discover pathophysiological principles that are involved in the development of hypertension both in experimental models and in humans. Compared to other approaches, including genomic studies the progress in the proteomic field is, however, slow despite the apparent potential of such studies [18]. In part this is due to proteomics being a relatively young area of research and the complexity not only of the proteome itself but also the proteomic platforms that require specialist skills to acquire and interpret the data. To large extent the slow progress is due to the complexity of hypertension itself. This has already been apparent in the first large genome-wide association study by the Wellcome Trust Case-Control Consortium [87] where the study of all other complex diseases resulted in significant and plausible hits whereas not a single genetic variant achieved genome-wide significance for hypertension. Only with changes in study designs [88] and further increased sample sizes [89] have genomic studies in hypertension evolved into a success story.

We do believe, however, that there is a degree of misconception when researchers try to find common pathological principles across patients with complex and multifactorial diseases. It is indeed the unique feature of such conditions that different factors contribute to different extent to an individual’s phenotype, and this contribution results from the interplay of genetic and environmental influences and can change over time. Rather than using omics technologies primarily to discover common pathophysiological features we would very much like to encourage researchers to use these techniques to precisely describe an individual’s molecular make-up at a given point in time. Proteomics is ideally placed to deliver on this task. A large number of proteins can be described in tissue samples or body fluids and individual signatures or fingerprints can be developed that will have the potential not only to define a specific disease phenotype but also to direct preventative and therapeutic measures in the spirit of precision medicine [90].

In current clinical practice, there is very little stratification of treatment in patients with hypertension and approaches often follow trial and error principles. Exceptions are the prescription of certain classes of antihypertensive drugs if they have additional benefits for co-existing conditions, e.g. ACE inhibitors to treat hypertension and prevent progression of kidney disease or beta blockers to treat hypertension and heart failure. Some guidelines, such as those by The National Institute for Health and Care Excellence (NICE) recommend the choice of initial antihypertensive therapy based on age and ethnicity – surrogates of renin levels and RAAS activity [8,91]. The recently published Prevention And Treatment of Hypertension With Algorithm-based therapy (PATHWAY) 2 trial demonstrated that treatment response to the mineralocorticoid receptor antagonist spironolactone can be predicted by activity of the RAAS as assessed by baseline renin levels [92]. We would expect that a more individualized definition of hypertension based on precise clinical phenotypes and supplemented by biomarker and omics data could provide a much more powerful tool to predict a patient’s risk and offer the right preventative and therapeutic approaches at minimal cost and with minimal side effects.

## 6. Expert commentary

Hypertension is a complex cardiovascular disorder. While in the rarer secondary forms of hypertension the predominant mechanisms that increase blood pressure are known, the more common primary form of hypertension results from the interaction of multiple pathogenetic principles, such as oxidative stress, endothelial dysfunction, inflammation and vascular stiffening.

Proteomic studies have the potential to discover new disease pathways and drug targets in hypertension despite the complexity of the disease. However, the complexity of hypertension and associated organ damage not only offers potential but is also associated with challenges for proteomic and other biomarker approaches. Therefore, studies in experimental models are currently the mainstay of proteomic research in hypertension. In our review we have focussed on two models of hypertension that illustrate the approaches in hypertension research. The Ang II infusion model represents a hypothesis-driven approach with a single primary cause of hypertension. It is particularly well suited to study the complex sequelae of a single pathogenetic factor. Other models that mimic endocrine forms of hypertension, renal hypertension, or reduced nitric oxide availability can complement this research and unravel both common and model-specific pathways. In contrast, the SHR is a model of genetically determined hypertension that is multifactorial in origin. In this and other models of primary hypertension proteomic techniques offer insight into the interaction of pathogenetic principles that lead to the phenotype of raised blood pressure.

The major hurdle at this point in time is, however, translation to human hypertension. While numerous studies are available in preclinical models there are few examples of validation of these findings in patients with hypertension. This is mainly due to the challenges related to tissue sampling in humans. Even if vascular, renal or cardiac tissues are available in some cohorts and have been successfully used for proteomic analyses, the absence of serial (longitudinal) samples makes it impossible to easily dissect cause and consequence of hypertension. While this challenge applies not only to proteomics but also to other biomarker approaches the complex nature and high costs of proteomics make it more difficult to compensate for the lack of serial samples by increasing the sample size of cross-sectional studies and adjusting for clinical variables including duration of hypertension.

In clinical studies, researchers are therefore currently focussing on consequences of hypertension including left ventricular hypertrophy and renal damage and use proteomic approaches to understand the transition from risk factor to organ damage both in the natural progression of hypertension and in patients on treatment. However, standardized approaches and much larger datasets than those currently available, together with translational approaches between experimental models and clinical practice will have to be used to achieve meaningful results. We have witnessed the enormous potential that genomics offered in complex diseases, such as hypertension, coronary artery disease and diabetes but this success is based on significant funding initiatives, international collaborations, standardized analysis methods, shared resources, and very large numbers of biosamples. In parallel to studies in well-defined preclinical models, the proteomic community needs to scale up its efforts in order to bring proteomic techniques to clinical research and practice.

## 7. Five-year view

The next five years will in all likeliness decide about the future of proteomics in hypertension research. The potential of proteomics in cardiovascular diseases has been recognized [18] but there are almost no large-scale programs that employ a standardized approach to the proteomics of hypertension. Without such efforts the field of proteomics is unlikely to make meaningful contributions to hypertension research, although it will continue to play an important role in other clinical conditions. We encourage the community to work together and develop large programs into the proteomics of hypertension to be ready for the era of precision medicine. We would like to propose a workplan that can be initiated within the next five years to make use of the opportunities that proteomic research offers for patients with hypertension but appreciate that it may take longer to fully deliver on this plan.

First, at the level of preclinical research we strongly believe that complex datasets, such as data resulting from proteomic studies cannot be analyzed in isolation but should be brought together with already existing data in the same and in other models. We have already witnessed the description of common molecular features across different models of hypertension and other cardiovascular diseases but call for stronger collaborative approaches to compare and integrate datasets. We expect that within the next five years there will be better understanding of model-specific pathogenetic factors of hypertension, as well as of shared pathways that are independent of the initial cause.

Second, clinical proteomic studies need to be better coordinated and larger in size so that reliable data can be obtained. Previous experiences with suboptimal study designs but also the general trend toward reduced availability of funding for biomedical research will ‘force’ the proteomic community to work together and to design impactful research. The potential that samples from clinical trials and large-scale repositories, such as the Framingham cohort or the UK Biobank offer will support large-scale proteomic approaches. We expect that in the first instance targeted proteomic studies will be conducted that offer large throughput at limited costs but also at low resolution. Such studies are indeed currently underway and will be reported within the next five years and support large-scale projects into untargeted proteomics in the longer term.

Third, and as a result of the above steps, we expect that proteomics will play a critical role in precision medicine in the future. In fact, the majority of biomarkers that are being used in clinical practice to stratify risk or diagnose disease are protein or peptide biomarkers (including e.g. cardiac troponins and natriuretic peptides) and there is no reason why a wider array of protein biomarkers should not be suited to better define an individual’s molecular make-up and thereby support decisions based on the principles of precision medicine.

Clearly the full potential of proteomics can only develop if data are integrated with precise clinical phenotypes, other omics data and other biomarkers. We will see further development of bioinformatics and systems biology to support data analysis. Again, this is not unrealistic to happen within the next five years as long as sufficient amounts of high-quality data will be generated. He rapid development of tools to analyze genomic data is an excellent example of the available opportunities. Only then proteomic research can translate into diagnostic and therapeutic approaches in patients with hypertension, targeting key tissues, including the endothelium, heart, and kidneys.

## Key issues

- Hypertension is a multifactorial condition that develops over a long period of time and is defined based on clinical criteria rather than on pathophysiological principles.
- Proteomic studies have been conducted to define molecular features of hypertension in experimental models and clinical studies. Proteins involved in angiogenesis, vasoconstriction, inflammation, and matrix degeneration have been repeatedly found in such studies.
- The majority of research studies focusses on proteomic signatures of hypertension-associated organ damage, such as heart failure and renal disease.
- The currently available studies are relatively small, not standardized, difficult to compare across each other and therefore cannot compete with much larger efforts in other omics disciplines, including genomics and metabolomics.
- Despite the challenges related to the disease itself and also to proteomics as a tool there is an enormous potential for proteomics to inform precision medicine approaches in the management of hypertension.

## References

[1] De Hert S. Physiology of hemodynamic homeostasis. Best Pract Res Clin Anaesthesiol. 2012;26(4):409–419. 23351228 10.1016/j.bpa.2012.10.004

[2] Singh M, Mensah GA, Bakris G. Pathogenesis and clinical physiology of hypertension. Cardiol Clin. 2010;28(4):545–559. 20937440 10.1016/j.ccl.2010.07.001

[3] Aubiniere-Robb L, Jeemon P, Hastie CE, et al Blood pressure response to patterns of weather fluctuations and effect on mortality. Hypertension. 2013;62(1):190–196. 23648702 10.1161/HYPERTENSIONAHA.111.00686

[4] Mancia G, Verdecchia P Clinical value of ambulatory blood pressure: evidence and limits. Circ Res. 2015;116(6):1034–1045. 25767288 10.1161/CIRCRESAHA.116.303755

[5] Moser M Historical perspectives on the management of hypertension. J Clin Hypertens (Greenwich). 2006;8(8 Suppl 2):15–20. 16894244 10.1111/j.1524-6175.2006.05836.x PMC8109518

[6] Franklin SS, Wong ND Hypertension and cardiovascular disease: contributions of the framingham heart study. Glob Heart. 2013;8(1):49–57. 25690263 10.1016/j.gheart.2012.12.004

[7] Mancia G, Fagard R, Narkiewicz K, et al 2013 ESH/ESC guidelines for the management of arterial hypertension: the task force for the management of arterial hypertension of the European society of hypertension (ESH) and of the European Society of Cardiology (ESC). J Hypertens. 2013;31(7):1281–1357. 23817082 10.1097/01.hjh.0000431740.32696.cc

[8] National Institute for Health and Care Excellence Clinical Guidance 127. [cited 2018 64 Available from: https://www.nice.org.uk/guidance/cg127 31841287

[9] James PA, Oparil S, Carter BL, et al 2014 evidence-based guideline for the management of high blood pressure in adults: report from the panel members appointed to the Eighth Joint National Committee (JNC 8). JAMA. 2014;311(5):507–520. 24352797 10.1001/jama.2013.284427

[10] Whelton PK, Carey RM, Aronow WS, et al 2017 ACC/AHA/AAPA/ABC/ACPM/AGS/APhA/ASH/ASPC/NMA/PCNA guideline for the prevention, detection, evaluation, and management of high blood pressure in adults: a report of the American College of Cardiology/American Heart Association task force on clinical practice guidelines. Hypertension. 2017;71(6):e13–e115. 29133356 10.1161/HYP.0000000000000065

[11] SPRINT Research Group, Wright JT Jr., Williamson JD, et al A randomized trial of intensive versus standard blood-pressure control. N Engl J Med 2015;373(22):2103–2116. 26551272 10.1056/NEJMoa1511939 PMC4689591

[12] Lewington S, Clarke R, Qizilbash N, et al Age-specific relevance of usual blood pressure to vascular mortality: a meta-analysis of individual data for one million adults in 61 prospective studies. Lancet. 2002;360(9349):1903–1913. 12493255 10.1016/s0140-6736(02)11911-8

[13] Padmanabhan S, Caulfield M, Dominiczak AF Genetic and molecular aspects of hypertension. Circ Res. 2015;116(6):937–959. 25767282 10.1161/CIRCRESAHA.116.303647

[14] Lawes CM, Vander Hoorn S, Rodgers A, et al Global burden of blood-pressure-related disease, 2001. Lancet. 2008;371(9623):1513–1518. 18456100 10.1016/S0140-6736(08)60655-8

[15] Mills KT, Bundy JD, Kelly TN, et al Global disparities of hypertension prevalence and control: a systematic analysis of population-based studies from 90 countries. Circulation. 2016;134(6):441–450. 27502908 10.1161/CIRCULATIONAHA.115.018912 PMC4979614

[16] Olsen MH, Angell SY, Asma S, et al A call to action and a lifecourse strategy to address the global burden of raised blood pressure on current and future generations: the Lancet commission on hypertension. Lancet. 2016;388(10060):2665–2712. 27671667 10.1016/S0140-6736(16)31134-5

[17] GBD 2013 mortality and causes of death collaborators. Global, regional, and national age-sex specific all-cause and cause-specific mortality for 240 causes of death, 1990-2013: a systematic analysis for the global burden of disease study 2013. Lancet. 2015;385(9963):117–171. 25530442 10.1016/S0140-6736(14)61682-2 PMC4340604

[18] Lindsey ML, Mayr M, Gomes AV, et al Transformative impact of proteomics on cardiovascular health and disease: a scientific statement from the American Heart Association. Circulation. 2015;132(9):852–872. 26195497 10.1161/CIR.0000000000000226

[19] Wilkins MR, Pasquali C, Appel RD, et al From proteins to proteomes: large scale protein identification by two-dimensional electrophoresis and amino acid analysis. Biotechnology (N Y). 1996;14(1):61–65. 9636313 10.1038/nbt0196-61

[20] You S-A, Wang QK Proteomics with two-dimensional gel electrophoresis and mass spectrometry analysis in cardiovascular research In: Wang QK, ed. Cardiovascular disease: methods and protocols volume 2: molecular medicine. Totowa, NJ: Humana Press; 2007 p. 15–26. 10.1385/1-59745-213-0:15 17085802

[21] Evans G, Wheeler C, Corbett J, et al Construction of HSC-2DPAGE: a two-dimensional gel electrophoresis database of heart proteins. Electrophoresis. 1997;18(3–4):471–479. 9150926 10.1002/elps.1150180322

[22] Wilm MS, Mann M Electrospray and Taylor-Cone theory, Dole’s beam of macromolecules at last? Int J Mass Spectrom Ion Process. 1994;136(2):167–180.

[23] Wisniewski JR, Zougman A, Nagaraj N, et al Universal sample preparation method for proteome analysis. Nat Meth. 2009;6(5):359–362. 10.1038/nmeth.1322 19377485

[24] Albalat A, Mullen W, Husi H, et al Tissue proteomics in vascular disease In: Touyz RM, Schiffrin EL eds. Hypertension: methods and protocols. New York, NY: Springer New York; 2017 p. 53–60. 10.1007/978-1-4939-6625-7_4 28116706

[25] Makarov A, Denisov E, Kholomeev A, et al Performance evaluation of a hybrid linear ion trap/Orbitrap mass spectrometer. Anal Chem. 2006;78(7):2113–2120. 16579588 10.1021/ac0518811

[26] Boja ES, Rodriguez H Mass spectrometry-based targeted quantitative proteomics: achieving sensitive and reproducible detection of proteins. Proteomics. 2012;12(8):1093–1110. 22577011 10.1002/pmic.201100387

[27] Chang C-T, Yang C-Y, Tsai F-J, et al Mass spectrometry-based proteomic study makes high-density lipoprotein a biomarker for atherosclerotic vascular disease. BioMed Res Int. 2015;2015:13. 10.1155/2015/164846 PMC4450224 26090384

[28] Cui L, Nithipatikom K, Campbell WB Simultaneous analysis of angiotensin peptides by LC-MS and LC-MS/MS: metabolism by bovine adrenal endothelial cells. Anal Biochem. 2007;369(1):27–33. 17681269 10.1016/j.ab.2007.06.045 PMC2754136

[29] Delles C, Schiffer E, von Zur Muhlen C et al. Urinary proteomic diagnosis of coronary artery disease: identification and clinical validation in 623 individuals. J Hypertens. 2010;28(11):2316–2322. 20811296 10.1097/HJH.0b013e32833d81b7

[30] Neisius U, Koeck T, Mischak H, et al Urine proteomics in the diagnosis of stable angina. BMC Cardiovasc Disord. 2016;16:70. 27095611 10.1186/s12872-016-0246-y PMC4837614

[31] Terzi F, Cambridge S An overview of advanced SILAC-labeling strategies for quantitative proteomics. Methods Enzymol. 2017;585:29–47. 28109435 10.1016/bs.mie.2016.09.014

[32] Delles C, Neisius U, Carty DM Proteomics in hypertension and other cardiovascular diseases. Ann Med. 2012;44(Suppl 1):S55–S64. 22713150 10.3109/07853890.2012.660494

[33] Goldblatt H Studies on experimental hypertension, V: the pathogenesis of experimental hypertension due to renal ischemia. Ann Intern Med. 1937;11:69–103.

[34] Kim S, Tokuyama M, Hosoi M, et al Adrenal and circulating renin-angiotensin system in stroke-prone hypertensive rats. Hypertension. 1992;20(3):280–291. 1516946 10.1161/01.hyp.20.3.280

[35] Skeggs LT Jr., Kahn JR, Shumway NP The preparation and function of the hypertensin-converting enzyme. J Exp Med. 1956;103(3):295–299. 13295487 10.1084/jem.103.3.295 PMC2136590

[36] Kim S, Ohta K, Hamaguchi A, et al Angiotensin II induces cardiac phenotypic modulation and remodeling in vivo in rats. Hypertension. 1995;25(6):1252–1259. 7768570 10.1161/01.hyp.25.6.1252

[37] Schorb W, Booz GW, Dostal DE, et al Angiotensin II is mitogenic in neonatal rat cardiac fibroblasts. Circ Res. 1993;72(6):1245–1254. 8495553 10.1161/01.res.72.6.1245

[38] Simon G, Abraham G, Cserep G Pressor and subpressor angiotensin II administration. Two experimental models of hypertension. Am J Hypertens. 1995;8(6):645–650. 7662251 10.1016/0895-7061(95)00047-S

[39] Kawada N, Imai E, Karber A, et al A mouse model of angiotensin II slow pressor response: role of oxidative stress. J Am Soc Nephrol. 2002;13(12):2860–2868. 12444204 10.1097/01.asn.0000035087.11758.ed

[40] Li XC, Zhuo JL Phosphoproteomic analysis of AT1 receptor-mediated signaling responses in proximal tubules of angiotensin II-induced hypertensive rats. Kidney Int. 2011;80(6):620–632. 21697807 10.1038/ki.2011.161 PMC3164930

[41] Brooks HL, Allred AJ, Beutler KT, et al Targeted proteomic profiling of renal Na(+) transporter and channel abundances in angiotensin II type 1a receptor knockout mice. Hypertension. 2002;39(2 Pt 2):470–473. 11882592 10.1161/hy02t2.102959

[42] Gebhard S, Steil L, Peters B, et al Angiotensin II-dependent hypertension causes reversible changes in the platelet proteome. J Hypertens. 2011;29(11):2126–2137. 21946693 10.1097/HJH.0b013e32834b1991

[43] Ayyadevara S, Mercanti F, Wang X, et al Age- and hypertension-associated protein aggregates in mouse heart have similar proteomic profiles. Hypertension. 2016;67(5):1006–1013. 26975704 10.1161/HYPERTENSIONAHA.115.06849 PMC4833546

[44] Finckenberg P, Eriksson O, Baumann M, et al Caloric restriction ameliorates angiotensin II-induced mitochondrial remodeling and cardiac hypertrophy. Hypertension. 2012;59(1):76–84. 22068868 10.1161/HYPERTENSIONAHA.111.179457

[45] Gao BB, Stuart L, Feener EP Label-free quantitative analysis of one-dimensional PAGE LC/MS/MS proteome: application on angiotensin II-stimulated smooth muscle cells secretome. Mol Cell Proteomics. 2008;7(12):2399–2409. 18676994 10.1074/mcp.M800104-MCP200 PMC2596345

[46] McKinney CA, Fattah C, Loughrey CM, et al Angiotensin-(1-7) and angiotensin-(1-9): function in cardiac and vascular remodelling. Clin Sci (Lond). 2014;126(12):815–827. 24593683 10.1042/CS20130436

[47] Verano-Braga T, Schwammle V, Sylvester M, et al Time-resolved quantitative phosphoproteomics: new insights into Angiotensin-(1-7) signaling networks in human endothelial cells. J Proteome Res. 2012;11(6):3370–3381. 22497526 10.1021/pr3001755

[48] Wang HJ, Chen SF, Lo WY Identification of cofilin-1 induces G0/G1 arrest and autophagy in angiotensin-(1-7)-treated human aortic endothelial cells from iTRAQ quantitative proteomics. Sci Rep. 2016;6:35372. 27748441 10.1038/srep35372 PMC5066316

[49] Bamburg JR, McGough A, Ono S Putting a new twist on actin: ADF/cofilins modulate actin dynamics. Trends Cell Biol. 1999;9(9):364–370. 10461190 10.1016/s0962-8924(99)01619-0

[50] Wang W, Eddy R, Condeelis J The cofilin pathway in breast cancer invasion and metastasis. Nat Rev Cancer. 2007;7(6):429–440. 17522712 10.1038/nrc2148 PMC4270061

[51] Okamoto K, Aoki K Development of a strain of spontaneously hypertensive rats. Jpn Circ J. 1963;27:282–293. 13939773 10.1253/jcj.27.282

[52] Doggrell SA, Brown L Rat models of hypertension, cardiac hypertrophy and failure. Cardiovasc Res. 1998;39(1):89–105. 9764192 10.1016/s0008-6363(98)00076-5

[53] Koh-Tan HH, Dashti M, Wang T, et al Dissecting the genetic components of a quantitative trait locus for blood pressure and renal pathology on rat chromosome 3. J Hypertens. 2017;35(2):319–329. 27755386 10.1097/HJH.0000000000001155 PMC5214373

[54] Nabika T, Ohara H, Kato N, et al The stroke-prone spontaneously hypertensive rat: still a useful model for post-GWAS genetic studies? Hypertens Res. 2012;35(5):477–484. 22399095 10.1038/hr.2012.30

[55] Atanur SS, Birol I, Guryev V, et al The genome sequence of the spontaneously hypertensive rat: analysis and functional significance. Genome Res. 2010;20(6):791–803. 20430781 10.1101/gr.103499.109 PMC2877576

[56] Hubner N, Wallace CA, Zimdahl H, et al Integrated transcriptional profiling and linkage analysis for identification of genes underlying disease. Nat Genet. 2005;37(3):243–253. 15711544 10.1038/ng1522

[57] Pravenec M, Kurtz TW Recent advances in genetics of the spontaneously hypertensive rat. Curr Hypertens Rep. 2010;12(1):5–9. 20425152 10.1007/s11906-009-0083-9 PMC2821617

[58] Jin X, Xia L, Wang LS, et al Differential protein expression in hypertrophic heart with and without hypertension in spontaneously hypertensive rats. Proteomics. 2006;6(6):1948–1956. 16485256 10.1002/pmic.200500337

[59] Lee CK, Han JS, Won KJ, et al Diminished expression of dihydropteridine reductase is a potent biomarker for hypertensive vessels. Proteomics. 2009;9(21):4851–4858. 19743417 10.1002/pmic.200800973

[60] Bendall JK, Douglas G, McNeill E, et al Tetrahydrobiopterin in cardiovascular health and disease. Antioxid Redox Signal. 2014;20(18):3040–3077. 24294830 10.1089/ars.2013.5566 PMC4038990

[61] Hatziioanou D, Barkas G, Critselis E, et al Chloride intracellular channel 4 overexpression in the proximal tubules of kidneys from the spontaneously hypertensive rat: insight from proteomic analysis. Nephron. 2018;138(1):60–70. 29131056 10.1159/000479169

[62] Feng H, Li H, Zhang D, et al Aortic wall proteomic analysis in spontaneously hypertensive rats with a blood pressure decrease induced by 6-week load-free swimming. Biomed Rep. 2015;3(5):681–686. 26405545 10.3892/br.2015.488 PMC4534868

[63] Low TY, van Heesch S, van den Toorn H, et al Quantitative and qualitative proteome characteristics extracted from in-depth integrated genomics and proteomics analysis. Cell Rep. 2013;5(5):1469–1478. 24290761 10.1016/j.celrep.2013.10.041

[64] Manakov D, Ujcikova H, Pravenec M, et al Alterations in the cardiac proteome of the spontaneously hypertensive rat induced by transgenic expression of CD36. J Proteomics. 2016;145:177–186. 27132684 10.1016/j.jprot.2016.04.041

[65] Fields LE, Burt VL, Cutler JA, et al The burden of adult hypertension in the United States 1999 to 2000: a rising tide. Hypertension. 2004;44(4):398–404. 15326093 10.1161/01.HYP.0000142248.54761.56

[66] Carty DM, Schiffer E, Delles C Proteomics in hypertension. J Hum Hypertens. 2013;27(4):211–216. 22874797 10.1038/jhh.2012.30

[67] Currie G, Delles C The future of “omics” in hypertension. Can J Cardiol. 2017;33(5):601–610. 28161100 10.1016/j.cjca.2016.11.023 PMC5417769

[68] Argiles A, Rodriguez M, Ortiz A Is plasma proteomics able to provide alternative paths to better understand hypertension? Hypertension. 2017;70(2):250–252. 28652468 10.1161/HYPERTENSIONAHA.117.09049

[69] Gajjala PR, Jankowski V, Heinze G, et al Proteomic-biostatistic integrated approach for finding the underlying molecular determinants of hypertension in human plasma. Hypertension. 2017;70(2):412–419. 28652472 10.1161/HYPERTENSIONAHA.116.08906

[70] Matafora V, Zagato L, Ferrandi M, et al Quantitative proteomics reveals novel therapeutic and diagnostic markers in hypertension. BBA Clin. 2014;2:79–87. 26672470 10.1016/j.bbacli.2014.10.001 PMC4633972

[71] Johansson M, Ricci F, Aung N, et al Proteomic profiling for cardiovascular biomarker discovery in orthostatic hypotension. Hypertension. 2018;71(3):465–472. 29295851 10.1161/HYPERTENSIONAHA.117.10365

[72] Kuznetsova T, Mischak H, Mullen W, et al Urinary proteome analysis in hypertensive patients with left ventricular diastolic dysfunction. Eur Heart J. 2012;33(18):2342–2350. 22789915 10.1093/eurheartj/ehs185 PMC3705161

[73] Zhang Z, Staessen JA, Thijs L, et al Left ventricular diastolic function in relation to the urinary proteome: a proof-of-concept study in a general population. Int J Cardiol. 2014;176(1):158–165. 25065337 10.1016/j.ijcard.2014.07.014 PMC4155932

[74] Brown CE, McCarthy NS, Hughes AD, et al Urinary proteomic biomarkers to predict cardiovascular events. Proteomics Clin Appl. 2015;9(5–6):610–617. 25786980 10.1002/prca.201400195

[75] Petriz BA, Franco OL Effects of hypertension and exercise on cardiac proteome remodelling. Biomed Res Int. 2014;2014:634132. 24877123 10.1155/2014/634132 PMC4022191

[76] Pena MJ, Jankowski J, Heinze G, et al Plasma proteomics classifiers improve risk prediction for renal disease in patients with hypertension or type 2 diabetes. J Hypertens. 2015;33(10):2123–2132. 26237555 10.1097/HJH.0000000000000685

[77] Baldan-Martin M, Mourino-Alvarez L, Gonzalez-Calero L, et al Plasma molecular signatures in hypertensive patients with renin-angiotensin system suppression: new predictors of renal damage and de novo albuminuria indicators. Hypertension. 2016;68(1):157–166. 27217411 10.1161/HYPERTENSIONAHA.116.07412

[78] Martin-Lorenzo M, Gonzalez-Calero L, Martinez PJ, et al Immune system deregulation in hypertensive patients chronically RAS suppressed developing albuminuria. Sci Rep. 2017;7(1):8894. 28827575 10.1038/s41598-017-09042-2 PMC5566220

[79] Gonzalez-Calero L, Martinez PJ, Martin-Lorenzo M, et al Urinary exosomes reveal protein signatures in hypertensive patients with albuminuria. Oncotarget. 2017;8(27):44217–44231. 28562335 10.18632/oncotarget.17787 PMC5546475

[80] Zhang W, Chen X, Yan Z, et al Detergent-insoluble proteome analysis revealed aberrantly aggregated proteins in human preeclampsia placentas. J Proteome Res. 2017;16(12):4468–4480. 28965414 10.1021/acs.jproteome.7b00352

[81] Jin X, Xu Z, Cao J, et al Proteomics analysis of human placenta reveals glutathione metabolism dysfunction as the underlying pathogenesis for preeclampsia. Biochim Biophys Acta. 2017;1865(9):1207–1214. 28705740 10.1016/j.bbapap.2017.07.003

[82] Mary S, Kulkarni MJ, Malakar D, et al Placental proteomics provides insights into pathophysiology of pre-eclampsia and predicts possible markers in plasma. J Proteome Res. 2017;16(2):1050–1060. 28030762 10.1021/acs.jproteome.6b00955

[83] Ma K, Jin H, Hu R, et al A proteomic analysis of placental trophoblastic cells in preeclampsia-eclampsia. Cell Biochem Biophys. 2014;69(2):247–258. 24343450 10.1007/s12013-013-9792-4

[84] Myers JE, Tuytten R, Thomas G, et al Integrated proteomics pipeline yields novel biomarkers for predicting preeclampsia. Hypertension. 2013;61(6):1281–1288. 23547239 10.1161/HYPERTENSIONAHA.113.01168

[85] Chen G, Zhang Y, Jin X, et al Urinary proteomics analysis for renal injury in hypertensive disorders of pregnancy with iTRAQ labeling and LC-MS/MS. Proteomics Clin Appl. 2011;5(5–6):300–310. 21538910 10.1002/prca.201000100

[86] Carty DM, Siwy J, Brennand JE, et al Urinary proteomics for prediction of preeclampsia. Hypertension. 2011;57(3):561–569. 21199994 10.1161/HYPERTENSIONAHA.110.164285

[87] Wellcome Trust Case Control Consortium Genome-wide association study of 14,000 cases of seven common diseases and 3,000 shared controls. Nature. 2007;447(7145):661–678. 17554300 10.1038/nature05911 PMC2719288

[88] Padmanabhan S, Melander O, Johnson T, et al Genome-wide association study of blood pressure extremes identifies variant near UMOD associated with hypertension. PLoS Genet. 2010;6(10):e1001177. 21082022 10.1371/journal.pgen.1001177 PMC2965757

[89] Surendran P, Drenos F, Young R, et al Trans-ancestry meta-analyses identify rare and common variants associated with blood pressure and hypertension. Nat Genet. 2016;48(10):1151–1161. 27618447 10.1038/ng.3654 PMC5056636

[90] Dominiczak A, Delles C, Padmanabhan S Genomics and precision medicine for clinicians and scientists in hypertension. Hypertension. 2017;69(4):e10–e13. 28193712 10.1161/HYPERTENSIONAHA.116.08252

[91] Buhler FR, Burkart F, Lutold BE, et al Antihypertensive beta blocking action as related to renin and age: a pharmacologic tool to identify pathogenetic mechanisms in essential hypertension. Am J Cardiol. 1975;36(5):653–669. 242209 10.1016/0002-9149(75)90168-x

[92] Williams B, MacDonald TM, Morant S, et al Spironolactone versus placebo, bisoprolol, and doxazosin to determine the optimal treatment for drug-resistant hypertension (PATHWAY-2): a randomised, double-blind, crossover trial. Lancet. 2015;386(10008):2059–2068. 26414968 10.1016/S0140-6736(15)00257-3 PMC4655321

